# Effects of Emotional Contexts on Cerebello-Thalamo-Cortical Activity during Action Observation

**DOI:** 10.1371/journal.pone.0075912

**Published:** 2013-09-26

**Authors:** Viridiana Mazzola, Patrik Vuilleumier, Valeria Latorre, Annamaria Petito, Vittorio Gallese, Teresa Popolizio, Giampiero Arciero, Guido Bondolfi

**Affiliations:** 1 Swiss Center for Affective Sciences (CISA), University of Geneva, Geneva, Switzerland; 2 Laboratory for Behavioral Neurology and Imaging of Cognition, Department of Neurology, University Hospital & Department of Neuroscience, Medical School University of Geneva, Geneva, Switzerland; 3 Institute of Post-Rationalist Psychology (IPRA), Rome, Italy; 4 Institute of Psychiatry and Clinical Psychology, Department of Medical Sciences, University of Foggia, Foggia, Italy; 5 Department of Neuroscience, Section of Physiology University of Parma & Italian Institute of Technology (IIT) Brain Center for Social and Motor Cognition, Parma, Italy; 6 Department of Neuroradiology, “Casa Sollievo della Sofferenza” IRCCSS, San Giovanni Rotondo (FG), Foggia, Italy; 7 Department of Mental Health and Psychiatry, University Hospital of Geneva, Geneva, Switzerland; Medical University of Vienna, Austria

## Abstract

Several studies investigated the neural and functional mechanisms underlying action observation in contexts with objects. However, actions seen in everyday life are often embedded in emotional contexts. The neural systems integrating emotion cues in action observation are still poorly understood. Previous findings suggest that the processing of both action and emotion information recruits motor control areas within the cerebello-thalamo-cortical pathways. It is therefore hard to determine whether social emotional contexts influence action processing via a direct modulation of motor representations coding for the observed action or via the affective state and implicit motor preparedness elicited in observers in response to emotional contexts. Here we designed a novel fMRI task to identify neural networks engaged by the affective appraisal of a grasping action seen in two different emotional contexts, while keeping the action kinematics constant. Results confirmed that observing the same acts of grasping but in different emotional contexts modulated activity in supplementary motor area, ventrolateral thalamus, anterior cerebellum. Moreover, changes in functional connectivity between left supplementary motor area and parahippocampus in different emotional contexts suggested a direct neural pathway through which emotional contexts may drive the neural motor system. Taken together, these findings shed new light on the malleability of motor system as a function of emotional contexts.

## Introduction

A key challenge of social and affective neuroscience is to understand how neural systems control behaviors in social and emotional situations [1-3]. Many studies in the field of social behavior have focused on action observation as the recognition of grasping hand actions performed in context containing objects [4-7]. Contextual influences are important in so far as they refer to the ways by which the perceptual qualities of a local feature are affected by surrounding scene elements and the way in which global scene characteristics affect the responses of neurons to local features. Therefore, a serious limitation of action recognition studies with respect of social behaviors, however, is that they generally do not consider that observed actions are often embedded in an emotional context. Far too little attention has been paid to the neural underpinnings of social behavior that occurs in response to an action seen in an emotional context. In everyday life, we constantly find ourselves to be affectively engaged in dynamic interactions with other people who display emotions together with their action or intention to act.

However, several findings point to a neural link between emotion and movement control. Indeed, emotions can be characterized by particular action tendencies that are manifested or inhibited according to the emotion type and context [8]. Using transcranial magnetic stimulation (TMS), greater motor cortex excitability was found in the supplementary motor area (SMA) in response to emotionally arousing positive and negative images than to neutral, non-emotional images [9,10]. Subsequent studies have extended the involved networks down to the brainstem and spinal cord. For instance, photographs of faces expressing fear elicit greater excitability in the corticospinal tract of the observer, as compared with faces expressing happiness or no emotion (i.e., neutral faces) [11-13] as well as negative scenes compared to positive scenes [14]. Similar corticospinal excitability increases have been detected in response to negative emotional scenes [15]. As pointed out by these authors, this greater responsiveness of motor network suggests that emotional reactions often include some form of action preparedness, engaging areas directly involved in movement control such as the cerebello-thalamo-cortical pathways [16-20].

Given these results, it is reasonable to hypothesize that emotional contexts can modulate neural activity in those areas directly involved in movement control. Fearful expressions presented in faces simultaneously with motor execution or inhibition cues can produce significant changes in levels of activity within primary motor cortex and premotor areas, including SMA [21,22]. There is also growing evidence that cerebellar functions are involved in emotion processing rather than engaged by purely motor parameters [23,24]. In addition, thalamic and subthalamic nuclei may play a key role in gating emotional information transfer between cortical and subcortical regions, thereby modulating motor commands at various stages [25-27].

Therefore, here we investigated whether any modulation of action observation by the emotional context would affect primary sensorimotor processes relying on a common cerebello-thalamo-cortical pathway necessary for voluntary movements. It is noteworthy that this distributed neural pathway is engaged in clinical phenomena that involve both emotions and sensorimotor processes, such as essential tremor associated with anxiety and depression disorders [28], stuttering [29,30], impulsivity [31] and even hysteria [32-35].

To test the effect of emotional contexts on the cerebello-thalamo-cortical pathway associated with action control and action recognition, we designed a novel fMRI task allowing us to assess the effects of affective appraisals on action observation in two different emotional contexts, while keeping action kinematics constant. Twenty-three participants underwent fMRI while watching two runs of video clips, each including one basic emotion (joy or anger) [36]. The video clips showed either a hand grasping an object on a table (grasping alone), a person with a neutral facial expression grasping an object on a table (neutral grasping), a person with a joyful or angry facial expression grasping an object on a table (joyful grasping/angry grasping), or the joyful or angry expression without any grasping (joyful face/angry face). All the objects employed were everyday objects. These videos were edited with the Blue Screen technique so as to superimpose faces with different emotional expressions (neutral, joyful, or angry) over the same trunk of a person grasping the objects on a table in front of her. To avoid confounding motor-related activation in the cerebello-thalamo-cortical pathway, no overt response was required. Instead, the participants were instructed to watch the stimuli carefully. To evaluate affective engagement, we asked participants after scanning to recognize each of the expressed emotions and rate their intensity. Our main hypothesis was that the activity of motor circuits within the cerebellar-thalamo-cortical pathway would be differentially driven by the emotional contexts associated with observed actions.

## Methods

### Ethics statement

The present study was approved by the Comitato Etico Indipendente Locale of the Azienda Ospedaliera “Ospedale Policlinico Consorziale” of Bari. Informed written consent was obtained from all participants before participation.

### Participants

Twenty-three participants were enrolled (12 females; mean age 29; standard deviation [SD] 7.5). Exclusion criteria included a history of drug or alcohol abuse, previous head trauma with loss of consciousness, pregnancy, and any significant medical or psychiatric conditions as evaluated with the Structured Clinical Interview (SCID). The present study was approved by the local institutional review board. Informed written consent was obtained from all participants before participation. Before the scanning session, each participant completed the State-Trait Anxiety Inventory (STAI) [37] to evaluate their current state of anxiety.

### fMRI experimental paradigm

Functional MRI sessions consisted of two successive scans with an event-related design. Each run included one emotion (joy or anger) and consisted of four experimental view conditions. All visual stimuli consisted of video clips of 1.7 sec. There were 4 different video conditions in each run, showing the following actions: the trunk with the arm grasping an object on a table (grasping alone), a person with a neutral facial expression grasping an object on a table (neutral grasping), a person with a joyful or angry facial expression grasping an object on a table (joyful grasping in the joyful run or angry grasping in the angry run), and a joyful or angry dynamic facial expression without any grasping action (joyful face in the joyful run or angry face or in the angry run). The recording and editing of videos were made with the Blue Screen technique in order to superimpose on the same trunk different emotional facial expressions. This procedure allowed us to obtain stimuli with the same kinematics of grasping behavior across different emotion conditions. Two professional actors, a female and a male, were enrolled as models for the videos. Both the actors gave written informed consent, as outlined in the PLOS consent form, to publication of their image. Two investigators reviewed the videotaped recordings and selected by consensus the picture frames conveying the appropriate intensity of anger and joy, based on Ekman and Friesen’s Facial Action Coding System (FACS) [38]. The objects to be grasped were commonly put on a table (phone, pen, keys, bottle, cup, glass, pencil case). The grasping gestures were equally executed with the right and left arm.

In each scanning run, 4 visual stimuli per gender per 8 conditions were repeated 5 times (total 160 visual stimuli) and presented in random order optimized by genetic algorithm toolbox [39]. Each stimulus was presented for 1700 ms, with an average 1810 ms interstimulus interval (ISI). 48 additional null events, each lasting 2700 ms, contributed to randomly jitter the stimulus onsets. Total scanning run time was about 19 minutes. The presentation order of the two runs was counterbalanced across subjects. Visual stimuli were displayed using the software Presentation 12 [www.neuro-bs.com].

To circumvent any motor interference, we used a passive viewing task and participants were instructed to remain still without performing any movement, and to avoid any imitation or mental imagery of the actions shown. After scanning, thirty video clips were presented to participants again. Half of the videos were the same as those shown during the scanning session. The other half (new videos) showed the same actors and scenarios but with different facial expressions and behaviors. Participants were asked to recognize which video they had seen or not. Next, they were also asked to recognize which kind of emotions (joy or anger) was displayed in twenty video clips previously seen during fMRI task, and to rate their intensity by using a computerized visual analogue scale (VAS) with target words ranging from “no intensity” to “extreme intensity”.

### fMRI data acquisition and analyses

Three-dimensional images were acquired using a T1-weighted SPGR sequence (TR/TE/NEX=25/3/1; flip angle 6°; matrix size 256×256; FOV 25×25 cm) with 124 sagittal slices (1.3 mm thick, in-plane resolution of 0.94×0.94). fMRI data were acquired on a 3T GE (General Electric, Milwaukee, WI) MRI scanner with a gradient-echo echo planar imaging (EPI) sequence and covered 26 interleaved axial slices (5 mm thick, 1mm gap), encompassing the entire cerebrum and the cerebellum (TR 2; FOV 24 cm; matrix, 64 x 64, a voxel size of 3.75x3.75x5 mm). For each scan, a total of 285 EPI volume images were acquired.

#### Preprocessing

Data were preprocessed and analyzed using statistical parametric mapping SPM8 (Wellcome Department of Cognitive Neurology, London, UK), implemented in MatLab 7.8 (MathWorks^TM^). A fixed-effect model at a single-subject level was performed to create images of parameter estimates, which were then entered into a second-level random-effects analysis. For each subject, functional images were first slice-timing corrected, using the middle slice acquired in time as a reference, and then spatially corrected for head movement, using a least-squares approach and six-parameter rigid body spatial transformations. High-resolution anatomical T1 images were coregistered with the realigned functional images to enable anatomical localization of the activations. The two runs were then concatenated. Structural and functional images were spatially normalized into a standardized anatomical framework using the default EPI template provided in SPM8, based on the averaged-brain of the Montreal Neurological Institute and approximating the normalized probabilistic spatial reference frame of Talairach and Tournoux [40]. Functional images were spatially smoothed with a three-dimensional Gaussian filter (10mm full-width at half-maximum). The time series was temporally filtered to eliminate contamination from slow drift of signals (high-pass filter, 128 s) and corrected for autocorrelations using the AR(1) model in SPM8.

#### Cerebellar normalization

We used a separate normalization process for data from the cerebellum. The registration between individuals and MNI space is suboptimal in the cerebellum when using a standard whole-brain normalization process [41]. Because cerebella vary relatively little between individuals compared with the cortical landmarks used for whole-brain normalization, it is possible to achieve a much better registration by normalizing the cerebella separately. Moreover, precise spatial registration is important because cerebellar structures are small relative to cortical structures. To this aim, we used the SUIT toolbox [41] for SPM8 allowing us to normalize each individual’s structural scan to an infratentorial template, and then used the resulting deformation maps to normalize the cerebellar sections of each person’s functional images. The SUIT toolbox has the additional advantage that coordinates can be adjusted from MNI space to the corresponding coordinates on the unnormalized Colin-27 brain, which is described anatomically in a cerebellar atlas. We used this feature to identify anatomical regions within the cerebellum.

#### Statistical analyses

We performed two parallel but identical statistical analyses on the functional data for the whole-brain and cerebellar normalized images. Four event-types were defined per subject per scanning run, corresponding to each condition of interest. In the joyful run, the condition of interest were: grasping alone, neutral grasping, joyful grasping, joyful face. In the angry run, the conditions were: grasping alone, neutral grasping, angry grasping, angry face. Then, eight contrast images corresponding to these conditions were created using one-sample t-tests in all subjects and then entered at the second level into a repeated-measures 2x4 ANOVA with non-sphericity correction (as implemented in SPM8). Paired t-tests were used to examine differences between the two run conditions. For whole-brain analyses the statistical threshold was P=0.05 FWE corrected with an extent threshold of 8 contiguous voxels. Because of our strong a priori hypothesis, results in SMA, VL thalamus and cerebellar anterior lobe (HIV-HV) were corrected for multiple comparisons with family-wise error correction at P < 0.05 applied on the activated clusters, with the volume of interest defined by the corresponding anatomical region in the WFU Pickatlas (http://fmri.wfubmc.edu/cms/software#PickAtlas)*.*

Specific contrasts were created to determine the direction of the difference identified in the ANOVA. Cerebral MNI coordinates were converted to the Talairach coordinate system by icbm2tal [http://brainmap.org/icbm2tal/]. Anatomic and Brodmann areas labeling of cerebral activated clusters was performed with the Talairach Daemon database [http://www.talairach.org/]. Anatomic labeling of cerebellar peak coordinates was performed using the SPM Anatomy Toolbox Version 1.7b [42]. For subsequent visual inspection of the results and illustration of the beta weights in graphic plots, regions-of-interest (ROIs) were defined as spheres with 6mm diameter centered on the peak voxel of the activated clusters. Parameter estimates of signal intensity in these ROIs were then obtained from first-level analyses of each participant.

A psychophysiological interaction (PPI) analysis was also performed to identify brain regions exhibiting a change in functional connectivity with our a priori hypothesized regions. For each individual we extracted the BOLD time-series from the voxels within a 5 mm sphere surrounding the activation peak of the seed regions (left SMA, left VL thalamus, right HV). A general linear model was computed using three regressors: a physiological regressor (the time-series in the ROI), a psychological regressor (angry context – joyful context), and a psychophysiological interaction term, calculated as the cross-product of the previous two terms. These time-series were then mean centered, high-pass filtered, and deconvolved. Subject-specific contrast images were then entered into a random effects analysis using two t-tests (p<0.05 FWE corrected as above).

In order to explore the potential relationship between brain regions differentially activated by the two emotional contexts and subjective ratings relative to joy and anger intensity (obtained in post scanning questionnaires), we performed correlation analyses on the corresponding data. Pearson’s correlation tests were computed using the parameter estimates of differential activity in clusters that exhibited a significant context by condition interaction, and the mean difference between joy and anger intensity in the post scanning ratings.

## Results

### Neuroimaging Results

Before examining the effects of emotion, we first verified that brain regions engaged by observing the motor act of grasping replicated the findings of previous studies [43-45]. The simple contrast of the grasping action alone vs baseline (pooled across both sessions) demonstrated highly significant activations in the bilateral inferior occipital gyrus/BA19, bilateral superior parietal lobule/BA7, and left precentral gyrus/BA6 (P<0.05 FWE corrected) (Table 1). In addition, the cerebellar analysis revealed significant activation in the right HVIIa crus I cerebellar lobe (P<0.05 FWE corrected) (see Table 1).

**Table 1:** One sample T-test Grasping alone p<0.05 FWE corrected, k=8.

**MNI coordinates**					
**Region**	**x**	**y**	**z**	**Ke**	**Z Scores**
Right Inferior Occipital Gyrus BA19	50	-74	-10	480	23.52
Left Inferior Occipital Gyrus BA19	-44	-78	-10	528	20.18
Left Superior Parietal Lobule BA7	-33	-60	55	46	5.73
Left Precentral Gyrus BA6	-52	0	45	24	5.55
Right Superior Parietal Lobule BA7	34	-52	50	55	5.36
Right Cerebellum HVIIa crus I	46	-74	-35	760	5.31

Likewise, the main effect of grasping observation (grasping alone > faces alone, pooled across both emotion sessions) demonstrated significant activations in the left and right inferior occipital gyrus/BA18-19, right superior parietal lobule, and middle frontal gyrus/BA6. Cerebellar increases were found in the left HVIIa crus I and right HVI (P<0.05 FWE corrected) (Table 2). Conversely, the main effect of face observation (faces alone > grasping alone) revealed significant activations restricted to visual areas, including the right middle temporal gyrus/BA22, and left medial lingual gyrus/BA18 (P<0.05 FWE corrected) (Table 2). Taken together, these results are consistent with previous studies on hand action observation [46-48].

**Table 2:** Comparison of Grasping alone vs Faces alone, p<0.05 FWE corrected, k=8.

**MNI coordinates**					
**Region**	**x**	**y**	**z**	**Ke**	**Z Scores**
**Grasping > Faces**					
Left Inferior Occipital Gyrus BA19	-48	-74	-10	953	15.91
Right Inferior Occipital Gyrus BA18	38	-86	0	740	13.66
Right Superior Parietal Lobule	16	-30	40	8	4.65
Left Middle Frontal Gyrus BA6	-22	8	55	8	4.51
Left Cerebellum HVIIa crus I	-34	-64	-33	182	3.56
Right Cerebellum HVI	34	-64	-21	680	4.02
**Faces > Grasping**					
Right Middle Temporal Gyrus BA22	53	-41	0	87	7.68
Left Medial Lingual Gyrus BA18	1	-90	10	8	5.01

To test the main effect of different emotion contexts, we then carried out a paired t-test comparing brain activity between the two emotion runs (across all stimulus conditions). The angry run revealed greater activation in the left inferior frontal gyrus (IFG)/BA 47 (pars orbitalis), right anterior cingulate cortex/BA33, and left superior medial frontal gyrus/BA8 (P<0.05 FWE corrected). Concerning cerebellar activity, the angry run activated the right lobule HVI, right lobule HVIIa, and left lobule HX (Table 3). In contrast, activations in the joyful compared to the angry run did not reach statistical significance.

**Table 3:** Comparison between Joy and Angry Runs, p<0.05 FWE corrected.

**MNI coordinates**					
**Region**	**x**	**y**	**z**	**Ke**	**Z Scores**
**Angry > Joyful run**					
Left Inferior Frontal Gyrus pars orbitalis BA47	-44	30	-5	14	5.23
Right Anterior Cingulate Cortex BA33	12	12	20	12	5.20
Left Superior Medial Frontal Gyrus BA8	1	53	35	8	4.85
Right Cerebellum HVI	30	-42	-37	6	4.39
Right Cerebellum HVIIa	36	-54	-57	6	4.34
Left Cerebellum HX	-28	-30	-47	5	4.28

**Figure 1:**
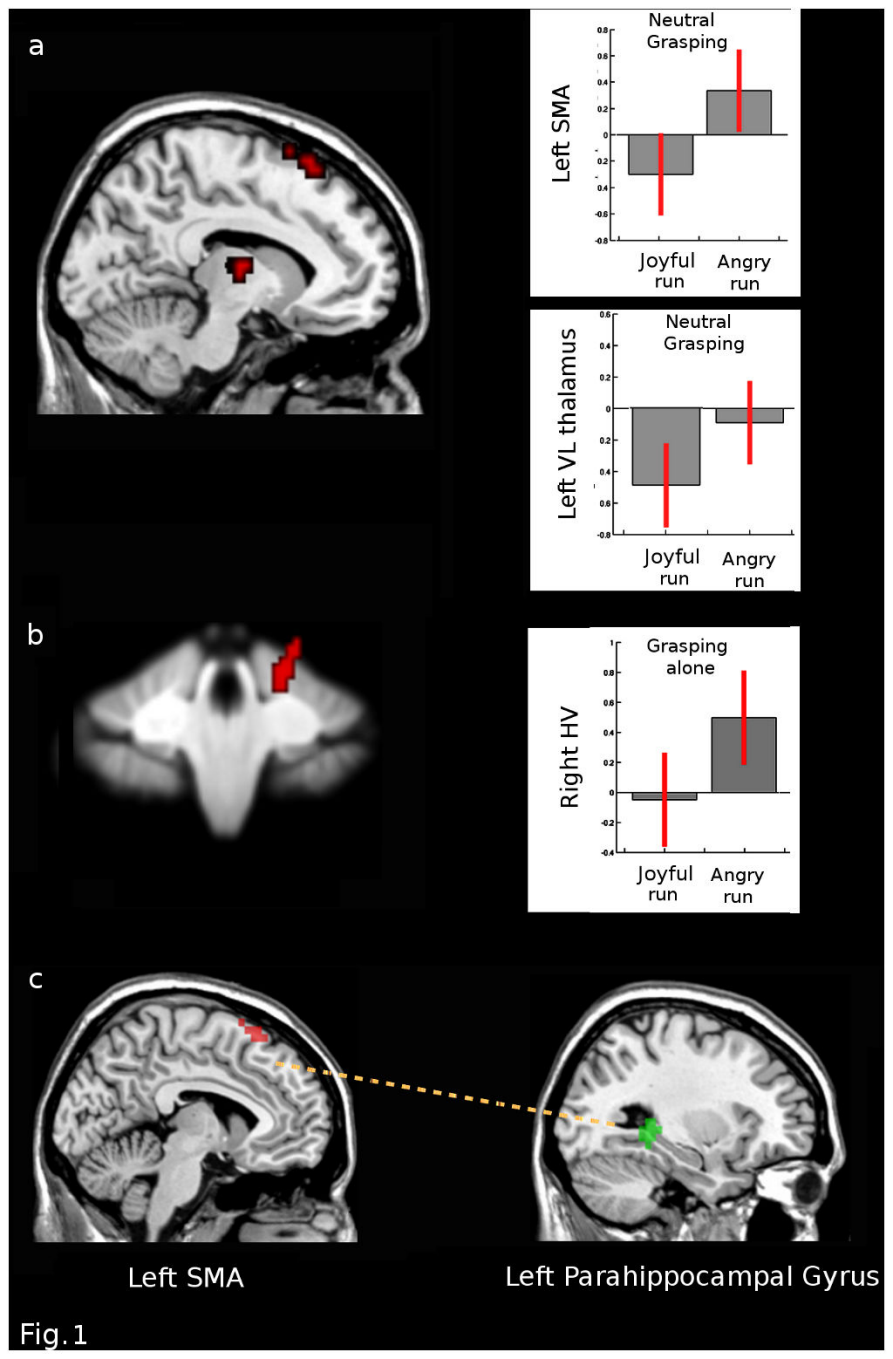
Interaction and PPI analyses between Grasping and Neutral Grasping across emotional contexts. BOLD response in terms of % signal change in the two emotional contexts: fMRI result for the 2-way interaction analyses in: **a**) the left SMA (peak coordinates x= -7, y= 27, z= 65, P<0.05 FWE corrected); the left VL thalamus (peak coordinates x= -14, y= -11, z= 10, P<0.05 FWE corrected). **b**) the right cerebellar lobule HV (peak coordinates x= 18, y= -38, z= -21, P<0.05 FWE corrected) (Mean±0.95 confidence intervals). **c**) PPI analysis: seed region in the left SMA (peak coordinates x= -7, y= 27, z= 65, P<0.05 FWE corrected); left parahippocampal gyrus/BA19 (peak coordinates x= -33, y= -41, z= -5, P<0.05 FWE corrected). Bars depict variance loadings and 90% confidence intervals.

We then verified our critical hypotheses. First, we examined whether the emotional context is associated with different neural responses during the observation of actions with the same kinematic in the key regions of interest. To this aim, we carried out an interaction analysis between grasping accompanied with a neutral face and grasping alone in the two different emotion runs (i.e. [neutral grasping – grasping alone] in angry runs vs. [neutral grasping – grasping alone] in joyful runs; and vice versa). Note that both grasping actions and neutral grasping were actually identical, and without emotional meaning by themselves, but they were seen in different emotion runs (intermingled with either angry or joyful faces). This interaction thus isolated any effect of emotion context on the processing of grasping actions, but unrelated to differences in face expression or grasp kinematics. Results demonstrated differential activity in the left SMA, left ventrolateral thalamus and right anterior cerebellar lobe HV (P<0.05 FWE corrected) (Table 4). Specific post hoc contrasts confirmed that the angry run elicited greater increases in the left SMA than the joyful run, specifically during the observation of grasping with the neutral face (Z=3.20) (Figure 1a); whereas the right anterior cerebellar lobe HV showed distinctive increases in the angry context when observing grasp alone, relative to other conditions (Z=2.30) (Figure 1b). On the other hand, the left ventrolateral thalamus was less engaged when processing the grasping action with a neutral face in the joyful run, relative to the angry run (Z= 3.06) (Figure 1a). Inspection of BOLD signal extracted from these clusters was consistent with the SPM data. There was no interaction effect showing greater responses in the joyful run.

**Table 4:** Interaction and PPI analyses between Grasping and Neutral Grasping across emotional contexts p< 0.05 FWE corrected.

**MNI coordinates**					
**Region**	**x**	**y**	**z**	**Ke**	**Z Scores**
Left SMA BA6	-7	27	65	21	3.74
Left ventrolateral Thalamus	-14	-11	10	48	3.40
Right Cerebellum HV	18	-38	-21	6	2.92
**PPI: seed in the left SMA**					
Left Parahippocampal Gyrus BA19	-33	-41	-5	8	3.71

Next, to explore possible changes in functional connectivity associated with these emotion context effects, we performed PPI analyses with seeds placed in each of the three ROIs showing these significant interactions. Only the PPI with a seed region in the left SMA survived the set threshold (p<0.05 FWE corrected) (Table 4). The left SMA exhibited a strong positive connectivity with left parahippocampal gyri/BA19 (PHG) selectively when the grasping action was seen with a neutral face in the angry run (as compared with the exact same neutral grasping action but seen in the joyful run) (Figure 1c).

### Postscanning behavioral results

The rate of recognition errors for each emotion type was very low (mean 1.77 [SD] 2.3) and did not differ between the two emotions (t= 1.2, p > 0.13 [SD] 1.8). The difference between the mean intensity rating for joy and anger was not statistically significant (t = -1.67, p > 0.11 [SD] 0.8), even though there was a trend in the expected direction (joy intensity mean= 3.45[SD] 0.8; anger intensity mean= 3.8[SD] 0.96).

**Figure 2:**
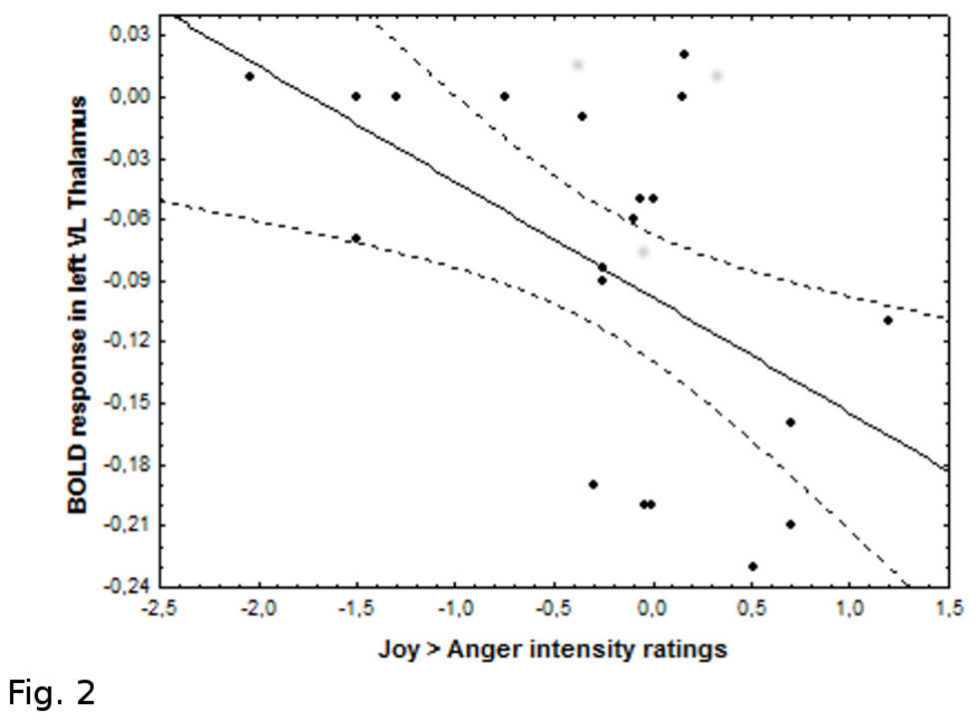
Postscanning behavioral results. Scatter plot showing the negative correlation between BOLD response within the left VL thalamic differential activity during neutral grasping in joyful and angry runs (joy > anger runs) and the mean difference between joy and anger intensity ratings (joy > anger ratings) (r = -0.54 p < 0.016).

However, we found different correlations between these affective post scanning ratings and activity in clusters that exhibited a significant context by condition interaction in SPM analysis (see above). There was a statistically significant negative correlation between differential activity of left ventrolateral thalamic during grasping in joyful and angry contexts (joy > anger runs) and the difference between joy and anger intensity perceived in the post-scanning judgments (joy > anger ratings) (r = -0.54, p < 0.016) (Figure 2). In other words, the more intense joy was perceived in faces by the participant, the less similar the angry and joy context effects were in the VL thalamus. On the other hand, there was no significant correlation between activity in SMA and post scanning intensity ratings (joy > anger: r = -0.17, p > 0.5; anger > joy: r = -0.03 p> 0.8).

## Discussion

In the present study, we investigated emotion-action interactions using a novel emotion contextual approach in which participants saw video clips of an actor grasping an object with different emotional expressions (neutral, joyful, or angry). Our design also allowed us to compare the same grasping with neutral faces but seen in different runs and intermingled with different emotion faces.

Our research question concerned whether the emotional contexts in which an action is seen affected the neural underpinnings of movement control. Context was manipulated by pairing the grasp action with a face expressing different emotions, or more purely by presenting neutral grasp actions intermingled with emotional faces. Consistent with our prediction, the interaction analysis concerning the same grasping action seen in different emotional contexts revealed greater activity in the left SMA and right cerebellar anterior lobe HV, selectively during the anger condition. Both SMA and cerebellum areas are critically involved in motor control [49]. In addition, the joyful context appeared to reduce activity in ventrolateral thalamus, which is not only another important relay within motor pathways [50,51], but also mediates the integration and coordination between distinct cortical areas recruited during voluntary movement [49].

Remarkably, these effects arose even though the face expression also remained neutral, while only the emotional context changed between blocks. Taken together, these findings suggest that observing acts of grasping with the same kinematics but in different emotional contexts can modulate specific brain circuits, and that such modulation does not involve areas associated with the recognition of goal-directed action such as premotor mirror neuron areas [4], but rather areas associated with the movements control and execution – namely, the cerebello-thalamo-cortical pathway [16–20,52]. Indeed, it is well known that the SMA is crucially involved in different aspects of preparation and inhibition of motor response [21,53–56]. The SMA is more engaged in non-conscious motor inhibition processes elicited by subliminal priming rather than in representing volitional planning or conscious goals [57-59], suggesting a potential role in automatic inhibition of partially activated responses. In our study, SMA increases were specific to the angry context, not seen in the joyful context. These results might be explained by the different emotional connotation of the grasping action that was conveyed in the angry context and that could thus directly modulate motor control processes. Consistent with these contentions, the differential activity in the VL thalamus (in angry vs joyful contexts) correlated with differences in perceived emotion intensity in the post scanning ratings (although stimuli were actually identical). These data therefore suggest that emotion contexts might directly modulate neural processes that are jointly associated with action readiness and recruited during action observation. Contrary to our expectations, however, we did not find a significant link between the SMA activity and emotional intensity ratings. It is possible that this negative result might be attributed to the inter-individual variability in the voluntary control of motor responses [57,59] or to less graded differences in the reactivity of SMA to emotional contexts.

These results also add support to some classic assumptions in the psychology of emotions. For instance, it is generally considered that negative emotions may narrow individuals’ momentary thought-action repertoires by calling forth specific action tendencies (e.g., attack, flee), whereas positive emotions broaden individuals’ momentary thought-action repertoires, prompting them to pursue a wider range of thoughts and actions than is typical (e.g., play, explore, savor, and integrate) [60]. According to the influential work of Frijda [61], anger involves readiness for a movement of opposition or hurting or “going against”, or of seeking to control someone else’s unwanted actions. On the other hand, joy involves a “free activation” signal for movement, in the form of an aimless, unasked-for readiness to engage in whatever interaction presents itself [61].

We also found greater positive functional coupling between the left SMA and the left parahippocampal gyrus (PHG) that was specific to observation of grasping in the angry context. This result suggests that the PHG might contribute to the influence of emotional context on motor activity. Indeed, this region has been implicated in contextual association processing during observation [62] and is sensitive to scene novelty [63], presumably supporting neural representations for the global context of episodes [64-67]. Other recent studies support the hypothesis that the PHG is associated with awareness of surrounding local space and responds to the basic sense of three-dimensional space [68,69]. The enhanced functional connectivity between SMA and PHG suggests a direct neural pathway through which the contextual emotional information could play a key role in guiding the motor system and representing action goals in space.

Some limitations of the present study have to be acknowledged. The first concerns the limited information on subjective emotional experience during different action observation conditions. Our VAS procedure used for post scanning rating might have been insufficient to detect a distinctive intensity of anger. This limitation is intrinsic to many self-reported measures. Even when respondents are doing their best to be forthright and insightful, their self-reports are subject to various sources of inaccuracy, including self-presentation and constraints on self-knowledge [70]. Moreover, appropriate designs and measures for experimental emotion elicitation are still issues far from settled [71]. When studying emotions and comparing them, there is always a possibility that some of the effects might be explained by a more general arousal factor. Notwithstanding these limitations, we believe it is difficult to interpret the fMRI results as explained by undetected arousal effects only. If this were the case, we would expect fMRI differences to be all in one direction (increase or decrease for anger relative to joy), whereas our data indicate changes in both directions. Furthermore, we did find significant correlations between emotion ratings and brain activity, which affected only some (i.e. thalamus) but not all activated areas (e.g. SMA). Still another limitation concerns the possibility of systematic but unseen movements of the hand while watching grasping videos. Despite instructions not to do so, such movements were not formally ruled out by direct camera recordings. However, we believe that systematic overt movements during this task would have produced differences in motor cortex activity in the contrast of all grasping conditions > facial expressions without any grasping. This contrast did not demonstrate any evidence of greater motor cortex activity, even at a more lenient statistical threshold (p< 0.005), suggesting that systematic movements of the hand did not take place while watching grasping videos.

Shifting from an action-based approach used in many previous studies [46,47,72,73] to an emotion-based approach as used here allows us to draw some new conclusions on the recognition of action in social emotional contexts. First, our findings show that action observation is affected by current emotional contexts even when action itself and its kinetic parameter are unchanged. Second, such effects of emotional context on action processing are at least in part mediated by a cerebello-thalamo-cortical network not related to the classic premotor mirror neuron areas [4-7], but rather to cortico-subcortical brain systems more directly associated with voluntary movement control. By eliciting differential activity in this system, emotional context might also contribute to motor flexibility and action readiness. Thus, in social interactions, the emotions perceived in others and/or conveyed by their actions are capable of modulating how motor circuits in the observer him/herself are emotionally engaged and prepared to (re)act – an effect that in turn exemplifies how emotion states can orient motor response [74-76] and generate a new set of possible actions [77,78].

## Supporting Information

Movie S1 **The actress grasps an object with an angry facial expression (angry grasping).**
(AVI)

Movie S2 **The actress grasps an object with a joyful facial expression (joyful grasping).**
(AVI)

Movie S3 **The actress grasps an object with a neutral facial expression (neutral grasping).**
(AVI)

Movie S4 **The actor grasps an object with an angry facial expression (angry grasping).**
(AVI)

Movie S5 **The actor grasps an object with a joyful facial expression (joyful grasping).**
(AVI)

Movie S6 **The actor grasps an object with a neutral facial expression (neutral grasping).**
(AVI)

## References

[1] Adolphs R (2010) Conceptual challenges and directions for social neuroscience. Neuron 65: 752-767. doi:10.1016/j.neuron.2010.03.006. PubMed: 20346753. 20346753 10.1016/j.neuron.2010.03.006 PMC2887730

[2] Frith C, Frith U (2010) Learning from Others: Introduction to the Special Review Series on Social Neuroscience. Neuron 65: 739-743. doi:10.1016/j.neuron.2010.03.015. PubMed: 20346750. 20346750 10.1016/j.neuron.2010.03.015

[3] Singer T (2012) The past, present, and future of social neuroscience: a European perspective. NeuroImage 61: 437–449. doi:10.1016/j.neuroimage.2012.01.109. PubMed: 22305955. 22305955 10.1016/j.neuroimage.2012.01.109

[4] Iacoboni M, Molnar-Szakacs I, Gallese V, Buccino G et al. (2005) Grasping the Intentions of Others with One’s Own Mirror Neuron System. PLOS Biol 3: 79. doi:10.1371/journal.pbio.0030079. PubMed: 15736981. 10.1371/journal.pbio.0030079 PMC1044835 15736981

[5] Keysers C, Gazzola V (2009) Expanding the mirror: vicarious activity for actions, emotions, and sensations. Curr Opin Neurobiol, 19: 666–71. doi:10.1016/j.conb.2009.10.006. PubMed: 19880311. 19880311 10.1016/j.conb.2009.10.006

[6] Rizzolatti G, Sinigaglia C (2010) The functional role of the parieto-frontal mirror circuit: interpretations and misinterpretations. Nat Rev Neurosci 11: 264-274. doi:10.1038/nrn2805. PubMed: 20216547. 20216547 10.1038/nrn2805

[7] Gallese V, Sinigaglia C (2011) What is so special about embodied simulation? Trends Cogn Sci 15: 512–519. PubMed: 21983148. 21983148 10.1016/j.tics.2011.09.003

[8] Frijda NH, Tcherkassof A (1995) Facial expressions as modes of action readiness. In: Russell JA Fernández-Dols J The Psychology of Facial Expression. Cambridge University Press.

[9] Oliveri M, Babiloni C, Filippi MM, Caltagirone C, Babiloni F et al. (2003) Influence of the supplementary motor area on primary motor cortex excitability during movements triggered by neutral or emotionally unpleasant visual cues. Exp Brain Res 149: 214–221. PubMed: 12610690. 12610690 10.1007/s00221-002-1346-8

[10] Hajcak G, Molnar C, George MS, Bolger K, Koola J, Nahas Z (2007) Emotion facilitates action: A transcranial magnetic stimulation study of motor cortex excitability during picture viewing. Psychophysiol 44: 91–97. doi:10.1111/j.1469-8986.2006.00487.x. PubMed: 17241144. 10.1111/j.1469-8986.2006.00487.x 17241144

[11] Schutter DJ, Hofman D, Van Honk J (2008) Fearful faces selectively increase corticospinal motor tract excitability: A transcranial magnetic stimulation study. Psychophysiology 45: 345–348. doi:10.1111/j.1469-8986.2007.00635.x. PubMed: 18221448. 18221448 10.1111/j.1469-8986.2007.00635.x

[12] Coombes SA, Tandonnet C, Fujiyama H, Janelle CM, Cauraugh JH et al. (2009) Emotion and motor preparation: a transcranial magnetic stimulation study of corticospinal motor tract excitability. Cogn Affect Behav Neurosci 9: 380–388. doi:10.3758/CABN.9.4.380. PubMed: 19897791. 19897791 10.3758/CABN.9.4.380

[13] van Loon AM, van den Wildenberg WP, van Stegeren AH, Ridderinkhof KR, Hajcak G (2010) Emotional stimuli modulate readiness for action: A transcranial magnetic stimulation study. Cogn Affect Behav Neurosci 10: 174-181. doi:10.3758/CABN.10.2.174. PubMed: 20498342. 20498342 10.3758/CABN.10.2.174

[14] Smith SD, Kornelsen J (2011) Emotion-dependent responses in spinal cord neurons: a spinal fMRI study. NeuroImage 58: 269-274. doi:10.1016/j.neuroimage.2011.06.004. PubMed: 21689762. 21689762 10.1016/j.neuroimage.2011.06.004

[15] Coelho CM, Lipp OV, Marinovic W, Wallis G, Riek S (2010) Increased corticospinal excitability induced by unpleasant visual stimuli. Neurosci Lett 481: 135–138. doi:10.1016/j.neulet.2010.03.027. PubMed: 20298754. 20298754 10.1016/j.neulet.2010.03.027

[16] Horne MK, Butler EG (1995) The role of the cerebello-thalamo-cortical pathway in skilled movement. Prog Neurobiol 46: 199-213. doi:10.1016/0301-0082(95)00002-D. PubMed: 7568913. 7568913

[17] Middleton FA, Strick PL (2001) Cerebellar projections to the prefrontal cortex of the primate. J Neurosci 21: 700-712. PubMed: 11160449. 11160449 10.1523/JNEUROSCI.21-02-00700.2001 PMC6763818

[18] Kelly RM, Strick PL (2003) Cerebellar loops with motor cortex and prefrontal cortex of a nonhuman primate. J Neurosci 23: 8432-8444. PubMed: 12968006. 12968006 10.1523/JNEUROSCI.23-23-08432.2003 PMC6740694

[19] Torriero S, Oliveri M, Koch G, Lo Gerfo E, Salerno S et al. (2011) Changes in cerebello-motor connectivity during procedural learning by actual execution and observation. J Cogn Neurosci 3: 338-348. PubMed: 20350172. 10.1162/jocn.2010.21471 20350172

[20] Carbon M, Argyelan M, Ghilardi MF, Mattis P, Dhawan V et al. (2011) Impaired sequence learning in dystonia mutation carriers: a genotypic effect. Brain 134: 1416-1427. doi:10.1093/brain/awr060. PubMed: 21515903. 21515903 10.1093/brain/awr060 PMC3097890

[21] Sagaspe P, Schwartz S, Vuilleumier P (2011) Fear and stop: A role for the amygdala in motor inhibition by emotional signals. NeuroImage 55: 1825-1835. doi:10.1016/j.neuroimage.2011.01.027. PubMed: 21272655. 21272655 10.1016/j.neuroimage.2011.01.027

[22] Pichon S, de Gelder B, Grèzes J (2012) Threat prompts defensive brain responses independently of attentional control. Cereb Cortex 22: 274-285. doi:10.1093/cercor/bhr060. PubMed: 21666127. 21666127 10.1093/cercor/bhr060

[23] Stoodley CJ, Schmahmann JD (2009) Functional topography in the human cerebellum: a meta-analysis of neuroimaging studies. NeuroImage 44: 489-501. doi:10.1016/j.neuroimage.2008.08.039. PubMed: 18835452. 18835452 10.1016/j.neuroimage.2008.08.039

[24] Baumann O, Mattingley JB (2012) Functional topography of primary emotion processing in the human cerebellum. NeuroImage 61: 805-811. doi:10.1016/j.neuroimage.2012.03.044. PubMed: 22465459. 22465459 10.1016/j.neuroimage.2012.03.044

[25] Tamietto M, Castelli L, Vighetti S, Perozzo P, Geminiani G et al. (2009) Unseen facial and bodily expressions trigger fast emotional reactions. Proc Natl Acad Sci U S A 106: 17661–17666. doi:10.1073/pnas.0908994106. PubMed: 19805044. 19805044 10.1073/pnas.0908994106 PMC2764895

[26] Tamietto M, de Gelder B (2010) Neural bases of the non-conscious perception of emotional signals. Nat Rev Neurosci 11: 697–709. doi:10.1038/nrg2844. PubMed: 20811475. 20811475 10.1038/nrn2889

[27] Pessoa L, Adolphs R (2010) Emotion processing and the amygdala: from a ‘low road’ to ‘many roads’ of evaluating biological significance. Nat Rev Neurosci 11: 773–783. doi:10.1038/nrn2920. PubMed: 20959860. 20959860 10.1038/nrn2920 PMC3025529

[28] Bermejo-Pareja F (2011) Essential tremor—a neurodegenerative disorder associated with cognitive defects? Nat. Rev Neurol 7: 273–282. doi:10.1038/nrneurol.2011.44. 10.1038/nrneurol.2011.44 21487422

[29] Chang SE, Horwitz B, Ostuni J, Reynolds R, Ludlow CL (2011) Evidence of left inferior frontal-premotor structural and functional connectivity deficits in adults who stutter. Cereb Cortex 21: 2507-2518. doi:10.1093/cercor/bhr028. PubMed: 21471556. 21471556 10.1093/cercor/bhr028 PMC3183422

[30] Neef NE, Jung K, Rothkegel H, Pollok B, Gudenberg AW, Paulus W et al. (2011) Right-shift for non-speech motor processing in adults who stutter. Cortex 47: 945-954. doi:10.1016/j.cortex.2010.06.007. PubMed: 20822768. 20822768 10.1016/j.cortex.2010.06.007

[31] Dalley JF, Everitt BJ, Robbins TW (2011) Impulsivity, Compulsivity, and Top-Down Cognitive Control. Neuron 69: 680-694. doi:10.1016/j.neuron.2011.01.020. PubMed: 21338879. 21338879 10.1016/j.neuron.2011.01.020

[32] Vuilleumier P, Chicherio C, Assal F, Schwartz S, Skusman D et al. (2001) Functional neuroanatomical correlates of hysterical sensorimotor loss. Brain 124: 1077-1090. doi:10.1093/brain/124.6.1077. PubMed: 11353724. 11353724 10.1093/brain/124.6.1077

[33] Cojan Y, Waber L, Carruzzo A, Vuilleumier P (2009) Motor inhibition in hysterical conversion paralysis. Neuroimage 47: 1026–1037. doi:10.1016/j.neuroimage.2009.05.023. PubMed: 19450695. 19450695 10.1016/j.neuroimage.2009.05.023

[34] Voon V, Brezing C, Gallea C, Ameli R, Roelofs K et al. (2010) Emotional stimuli and motor conversion disorder. Brain 133: 1526-1536. doi:10.1093/brain/awq054. PubMed: 20371508. 20371508 10.1093/brain/awq054 PMC2859149

[35] Edwards MJ, Adams RA, Brown H, Pareès I, Friston KJ (2012) A Bayesian account of ‘hysteria’. Brain. doi:10.1093/brain/aws129. 10.1093/brain/aws129 PMC3501967 22641838

[36] Ekman P (1999) Basic Emotions. In Dalgleish T Power M Handbook of Cognition and Emotion. Sussex, UK: John Wiley & Sons, Ltd.

[37] Spielberger C (1983) Manual for the State-Trait Anxiety Inventory. Palo Alto, CA: Consultant Psychologist Press.

[38] Ekman P, Friesen W (1976) Pictures of facial affect. Palo Alto, CA: Consulting Psychologist Press.

[39] Wager TD, Nichols TE (2003) Optimization of Experimental Design in fMRI: A General Framework Using a Genetic Algorithm. NeuroImage 18: 293-309. doi:10.1016/S1053-8119(02)00046-0. PubMed: 12595184. 12595184 10.1016/s1053-8119(02)00046-0

[40] Talairach J, Tournoux P (1998) Co-planar stereotaxic atlas of the human brain. New York: Thieme Verlag.

[41] Diedrichsen J, Balsters JH, Flavell J, Cussans E, Ramnani N (2009) A probabilistic MR atlas of the human cerebellum. Neuroimage 46: 39-46. doi:10.1016/j.neuroimage.2009.01.045. PubMed: 19457380. 19457380 10.1016/j.neuroimage.2009.01.045

[42] Eickhoff SB, Stephan KE, Mohlberg H, Grefkes C, Fink GR et al. (2005) A new SPM toolbox for combining probabilistic cytoarchitectonic maps and functional imaging data. NeuroImage 25: 1325-1335. doi:10.1016/j.neuroimage.2004.12.034. PubMed: 15850749. 15850749 10.1016/j.neuroimage.2004.12.034

[43] Castiello U (2005) The neuroscience of grasping. Nat Rev Neurosci. 6: 726-736. doi:10.1038/nrg1720. PubMed: 16100518. 16100518 10.1038/nrn1744

[44] Shmuelof L, Zohary E (2006) A mirror representation of others’ actions in the human anterior parietal cortex. J Neurosci 26: 9736-9742. doi:10.1523/JNEUROSCI.1836-06.2006. PubMed: 16988044. 16988044 10.1523/JNEUROSCI.1836-06.2006 PMC6674432

[45] Caspers S, Zilles K, Laird AR, Eickhoff SB (2010) ALE meta-analysis of action observation and imitation in the human brain. Neuroimage 50: 1148-1167. doi:10.1016/j.neuroimage.2009.12.112. PubMed: 20056149. 20056149 10.1016/j.neuroimage.2009.12.112 PMC4981639

[46] Grosbras MH, Paus T (2006) Brain networks involved in viewing angry hands or faces. Cereb Cortex 16: 1087-1096. PubMed: 16221928. 16221928 10.1093/cercor/bhj050

[47] Grosbras MH, Beaton S, Eickhoff SB (2011) Brain Regions Involved in Human Movement observation: A Quantitative Voxel-Based Meta-Analysis. Hum Br Mapp http://dx.doi.org/10.1002/hbm.21222. 10.1002/hbm.21222 PMC6869986 21391275

[48] Sabatinelli D, Fortune EE, Li Q, Siddiqui A, Krafft C, Oliver WT et al. (2011) Emotional perception: meta-analyses of face and natural scene processing. NeuroImage 54: 2524–2533. doi:10.1016/j.neuroimage.2010.10.011. PubMed: 20951215. 20951215 10.1016/j.neuroimage.2010.10.011

[49] Draganski B, Kherif F, Klöppel S, Cook PA, Alexander DC et al. (2008) Evidence for segregated and integrative connectivity patterns in the human Basal Ganglia. J Neurosci 28: 7143-7152. doi:10.1523/JNEUROSCI.1486-08.2008. PubMed: 18614684. 18614684 10.1523/JNEUROSCI.1486-08.2008 PMC6670486

[50] Johansen-Berg H, Behrens TEJ, Sillery E, Ciccarelli O, Thompson AJ et al. (2005) Functional–Anatomical Validation and Individual Variation of Diffusion Tractography-based Segmentation of the Human Thalamus. Cereb Cortex 15: 31-39. PubMed: 15238447. 15238447 10.1093/cercor/bhh105

[51] Spraker MB, Yu H, Corcos DM, Vaillancourt DE (2007) Role of Individual Basal Ganglia Nuclei in Force Amplitude Generation. J Neurophysiol 98: 821–834. doi:10.1152/jn.00239.2007. PubMed: 17567775. 17567775 10.1152/jn.00239.2007 PMC2367092

[52] Ramnani N (2006) The primate cortico-cerebellar system: anatomy and function. Nat Rev Neurosci 7: 511-522. doi:10.1038/nrn1953. PubMed: 16791141. 16791141 10.1038/nrn1953

[53] Nachev P, Kennard C, Husain M (2008) Functional role of the supplementary and pre-supplementary motor areas. Nat Rev Neurosci 9: 856-869. doi:10.1038/nrn2478. PubMed: 18843271. 18843271 10.1038/nrn2478

[54] Dinomais M, Minassian AT, Tuilier T, Delion M, Wilke M et al. (2009) Functional MRI comparison of passive and active movement: possible inhibitory role of supplementary motor area. Neuroreport 20: 1351-1355. doi:10.1097/WNR.0b013e328330cd43. PubMed: 19734813. 19734813 10.1097/WNR.0b013e328330cd43

[55] Boy F, Evans CJ, Edden RA, Singh KD, Husain M et al. (2010) Individual differences in subconscious motor control predicted by GABA concentration in SMA. Curr Biol 20: 1779-1785. doi:10.1016/j.cub.2010.09.003. PubMed: 20888227. 20888227 10.1016/j.cub.2010.09.003 PMC3128986

[56] Wardak C (2011) The role of the supplementary motor area in inhibitory control in monkeys and humans. J Neurosci 31: 5181-5183. doi:10.1523/JNEUROSCI.0006-11.2011.

[57] Sumner P, Nachev P, Morris P, Peters AM, Jackson SR et al. (2007) Human medial frontal cortex mediates unconscious inhibition of voluntary action. Neuron 54: 697–711. doi:10.1016/j.neuron.2007.05.016. PubMed: 17553420. 17553420 10.1016/j.neuron.2007.05.016 PMC1890004

[58] Sumner P, Husain M (2008) At the Edge of Consciousness: Automatic Motor Activation and Voluntary Control. Neuroscientist 14: 474-486. PubMed: 18356377. 18356377 10.1177/1073858408314435

[59] Boy F, Husain M, Singh KD, Sumner P (2010) Supplementary motor area activations in unconscious inhibition of voluntary action. Exp Brain Res 206: 441–448. doi:10.1007/s00221-010-2417-x. PubMed: 20871983. 20871983 10.1007/s00221-010-2417-x

[60] Fredrickson BL, Branigan C (2005) Positive emotions broaden the scope of attention and thought-action repertoires. Cogn Emot 19: 313-332. doi:10.1080/02699930441000238. PubMed: 21852891. 21852891 10.1080/02699930441000238 PMC3156609

[61] Frijda NH (1986) The emotions. Cambridge University Press.

[62] Kveraga K, Ghuman AS, Kassam KS, Aminoff EA, Hämäläinen MS et al. (2011) Early onset of neural synchronization in the contextual associations network. Proc Natl Acad Sci U S A 108: 3389-3394. doi:10.1073/pnas.1013760108. PubMed: 21300869. 21300869 10.1073/pnas.1013760108 PMC3044398

[63] Howard LR, Kumaran D, Ólafsdóttir HF, Spiers HJ (2011) Double dissociation between hippocampal and parahippocampal responses to object–background context and scene novelty. J Neurosci 31: 5253–5261. doi:10.1523/JNEUROSCI.6055-10.2011. PubMed: 21471360. 21471360 10.1523/JNEUROSCI.6055-10.2011 PMC3079899

[64] Bar M, Aminoff E (2003) Cortical analysis of visual context. Neuron 38: 347-358. doi:10.1016/S0896-6273(03)00167-3. PubMed: 12718867. 12718867 10.1016/s0896-6273(03)00167-3

[65] Davachi L (2006) Item, context and relational episodic encoding in humans. Curr Opin Neurobiol 16: 693-700. doi:10.1016/j.conb.2006.10.012. PubMed: 17097284. 17097284 10.1016/j.conb.2006.10.012

[66] Diana RA, Yonelinas AP, Ranganath C (2007) Imaging recollection and familiarity in the medial temporal lobe: a three-component model. Trends Cogn Sci 11: 379-386. doi:10.1016/j.tics.2007.08.001. PubMed: 17707683. 17707683 10.1016/j.tics.2007.08.001

[67] Eichenbaum H, Yonelinas AP, Ranganath C (2007) The medial temporal lobe and recognition memory. Annu Rev Neurosci 30: 123-152. doi:10.1146/annurev.neuro.30.051606.094328. PubMed: 17417939. 17417939 10.1146/annurev.neuro.30.051606.094328 PMC2064941

[68] Mullally SL, Maguire EA (2011) A New Role for the Parahippocampal Cortex in Representing Space. J Neurosci 31: 7441-7449. doi:10.1523/JNEUROSCI.0267-11.2011. PubMed: 21593327. 21593327 10.1523/JNEUROSCI.0267-11.2011 PMC3101571

[69] Wolbers T, Klatzky RL, Loomis JM, Wutte MG, Giudice NA (2011) Modality-Independent Coding of Spatial Layout in the Human Brain. Curr Biol 21: 984-989. doi:10.1016/j.cub.2011.04.038. PubMed: 21620708. 21620708 10.1016/j.cub.2011.04.038 PMC3119034

[70] Paulhus DL, Vazire S (2007) The Self-Report Method. In Robins RW Fraley RC Krueger RF Handbook of research methods in personality psychology. Guilford Press.

[71] Lench HC, Flores SA, Bench SW (2011) Discrete Emotions Predict Changes in Cognition, Judgment, Experience, Behavior, and Physiology: A Meta-Analysis of Experimental Emotion Elicitations. Psychol Bull, 137: 834–55. doi:10.1037/a0024244. PubMed: 21766999. 21766999 10.1037/a0024244

[72] de Gelder B (2006) Towards the neurobiology of emotional body language. Nat Rev Neurosci 7: 242-249. doi:10.1038/nrg1852. PubMed: 16495945. 16495945 10.1038/nrn1872

[73] Ferri F, Ebisch SJ, Costantini M, Salone A, Arciero G et al. (2013) Binding Action and Emotion in Social Understanding. Pone 8: 54091. doi:10.1371/journal.pone.0054091. PubMed: 23349792. 10.1371/journal.pone.0054091 PMC3547946 23349792

[74] Hinde RA (1985) Expression and negotiation. In Zivin G The development of expressive behaviour. New York: Academic Press pp 103-116.

[75] Hinde RA (1985) Was 'The expression of emotions' a misleading phrase? Anim Behav 33: 985-992. doi:10.1016/S0003-3472(85)80032-4.

[76] Griffiths PE, Scarantino A (2008) Emotions in the wild: The situated perspective on emotion. In Robbins P Aydede M The Cambridge Handbook of Situated Cognition. Cambridge University Press.

[77] Arciero G, Bondolfi G (2009) Selfhood, Identity and Personality. London: Wiley-Blackwell.

[78] Arciero G (1989) Evolutionary Epistemology and Scientific psychology. American Association of the Advancement of Science. San Francisco.

