# Correction: Effects of Emotional Contexts on Cerebello-Thalamo-Cortical Activity during Action Observation

**DOI:** 10.1371/annotation/73ee01f4-5b78-42c1-a4be-d48aa8ffd51d

**Published:** 2013-10-02

**Authors:** Viridiana Mazzola, Patrik Vuilleumier, Valeria Latorre, Annamaria Petito, Vittorio Gallese, Teresa Popolizio, Giampiero Arciero, Guido Bondolfi

There was an error in affiliations of the seventh author, Giampiero Arciero. This author's affiliations should only be:

Institute of Post-Rationalist Psychology (IPRA), Rome, Italy

Department of Mental Health and Psychiatry, University Hospital of Geneva, Geneva, Switzerland 

